# Evaluation of post-exposure prophylaxis practices to improve the cost-effectiveness of rabies control in human cases potentially exposed to rabies in southern Bhutan

**DOI:** 10.1186/s12879-020-4926-y

**Published:** 2020-03-06

**Authors:** Kinley Penjor, Nelly Marquetoux, Chendu Dorji, Kinley Penjor, Sithar Dorjee, Chencho Dorjee, P. D. Jolly, R. S. Morris, J. S. McKenzie

**Affiliations:** 1 School of Veterinary Science, Massey University, Auckland, New Zealand; 2 Khesar Gyalpo University of Medical Sciences of Bhutan, Thimphu, Bhutan; 3 Sarpang District Hospital, Department of Medical Services, Ministry of Health, Thimphu, Bhutan; 4 Present address: Vector-Borne Disease Control Program, Department of Public Health, Ministry of Health, Gelephu, Bhutan; 5 Regional Livestock Development Centre Tsimasham, Department of Livetsock, Ministry of Agriculture and Forest, Thimphu, Bhutan; 6 Bhutan Agriculture and Food Regulatory Authority, Ministry of Agriculture and Forest, Thimphu, Bhutan; 7 Faculty of Nursing and Public Health, Khesar Gyalpo University of Medical Sciences of Bhutan, Thimphu, Bhutan; 8 Morvet Ltd, Consultancy services in health risk management and food safety policy and programs, Masterton, New Zealand

**Keywords:** Bhutan, Clinical risk assessment, Neglected diseases, Post-exposure prophylaxis, Public health, Rabies

## Abstract

**Background:**

Rabies is endemic in southern Bhutan, associated with 1–2 human deaths and high post exposure prophylaxis (PEP) costs annually. Evaluation of clinicians’ management of human cases potentially exposed to rabies could contribute to improving PEP prescribing practices to both reduce unnecessary costs associated with PEP and reach the target of zero human deaths due to rabies by 2023.

**Methods:**

A cross-sectional survey of 50 clinicians’ management of human cases potentially exposed to rabies was conducted in 13 health centers in high-rabies-risk areas of Bhutan during February–March 2016.

**Results:**

Data were collected on clinicians’ management of 273 human cases potentially exposed to rabies. The 50 clinicians comprised health assistants or clinical officers (55%) and medical doctors (45%) with a respective median of 19, 21 and 2 years’ experience. There was poor agreement between clinicians’ rabies risk assessment compared with an independent assessment for each case based on criteria in the National Rabies Management Guidelines (NRMG). Of the 194 cases for which clinicians recorded a rabies risk category, only 53% were correctly classified when compared with the NRMG. Clinicians were more likely to underestimate the risk of exposure to rabies and appeared to prescribe PEP independently of their risk classification.. Male health assistants performed the most accurate risk assessments while female health assistants performed the least accurate. Clinicians in Basic Health Units performed less accurate risk assessments compared with those in hospitals.

**Conclusions:**

This study highlights important discrepancies between clinicians’ management of human cases potentially exposed to rabies and recommendations in the NRMG. In particular, clinicians were not accurately assessing rabies risk in potentially exposed cases and were not basing their PEP treatment on the basis of their risk assessment. This has significant implications for achieving the national goal of eliminating dog-mediated human rabies by 2030 and may result in unnecessary costs associated with PEP. Recommendations to improve clinician’s management of human cases potentially exposed to rabies include: reviewing and updating the NRMG, providing clinicians with regular and appropriately targeted training about rabies risk assessment and PEP prescription, and regularly reviewing clinicians’ practices.

## Background

Rabies remains a major public health threat in Asia with an estimated 39,000 deaths annually, mostly due to spill over from the canine reservoir [1]. Wider use of post-exposure prophylaxis (PEP) might reduce human mortalities in this region of the world [2]. On the other hand, the escalating cost of life-saving PEP represents a major burden to both national economies and families, mostly in poor rural communities [1, 3, 4].

In Bhutan, the number of reported animal rabies cases was stable in the decade 1996 to 2005 but increased during 2006 to 2008, mostly in 4 districts in southern Bhutan bordering India [5]. Maintenance of rabies in the canine reservoir in southern Bhutan was likely due to low coverage of dog vaccination programs. Since 2009, mass dog sterilization and vaccination programs contributed to a decline in the incidence of canine rabies [6]. However, the disease remains endemic in southern Bhutan and in some pockets in eastern Bhutan. All 13 human deaths due to rabies recorded in Bhutan between 2009 and 2017 were reported from southern districts where the estimated average annual incidence was 0.4 deaths/100,000 population [7, 8]. Past studies estimated that PEP intervention effectively averted about 15 human deaths annually in rabies endemic areas of Bhutan [9]. Hence the provision of free PEP is an important public health policy implemented by the Government of Bhutan.

PEP is a very significant on-going cost for the Government of Bhutan. Between 2009 and 2016, the annual number of reported dog bite victims presenting to health centers for PEP increased from 1000 to over 7000 [7]. Ministry of Health records show that about Nu. 9.2 million (USD 143,000) was spent for procurement of anti-rabies vaccines and immunoglobulin in the fiscal year 2016–17, representing approximately 6% of the essential medicines budget (Ministry of Health Medical procurement records 2016–17, unpublished). While PEP is an essential component of rabies control in Bhutan, inappropriate use of PEP can result in substantial costs to the health sector [10].

Implementation of PEP is guided by the Ministry of Health’s World Health Organization (WHO)-adapted National Rabies Management Guidelines 2014 (NRMG) which provides clinicians with criteria for categorizing rabies risk in potentially exposed people and recommends approaches to management of cases for the three risk categories. Prescription of Anti-Rabies Vaccine (ARV) is recommended for cases with a moderate or severe risk (NRMG Categories 2 and 3), while additional Rabies Immunoglobulin (RIG) administration is recommended for the highest risk category 3 exposures. To date there has been no evaluation of the management of human cases potentially exposed to rabies and the implementation of PEP in Bhutan. An evaluation would identify opportunities for improving the cost-effectiveness of PEP prescription to prevent human rabies cases whilst at the same time reducing wastage through unnecessary or inappropriate prescription of PEP in people that have a negligible risk of being exposed to the virus. Hence, the objective of this study was to evaluate clinicians’ management of human cases potentially exposed to rabies and PEP prescribing practices in the rabies-endemic areas of southern Bhutan. The results of this study were used to improve the cost-effectiveness with which this important rabies management policy is implemented in Bhutan to achieve the national goal of zero human rabies deaths in Bhutan by 2030 [11].

## Methods

We conducted a cross-sectional study to evaluate clinicians’ management and PEP prescription practices for human cases potentially exposed to rabies who sought treatment at health centers in southern Bhutan. The study protocol was approved by the Research Ethics Board of Health (REBH) under the Ministry of Health (Approval ref.no. *REBH/Approval/2016/001).*

### Study sites and participants

**Fig. 1:**
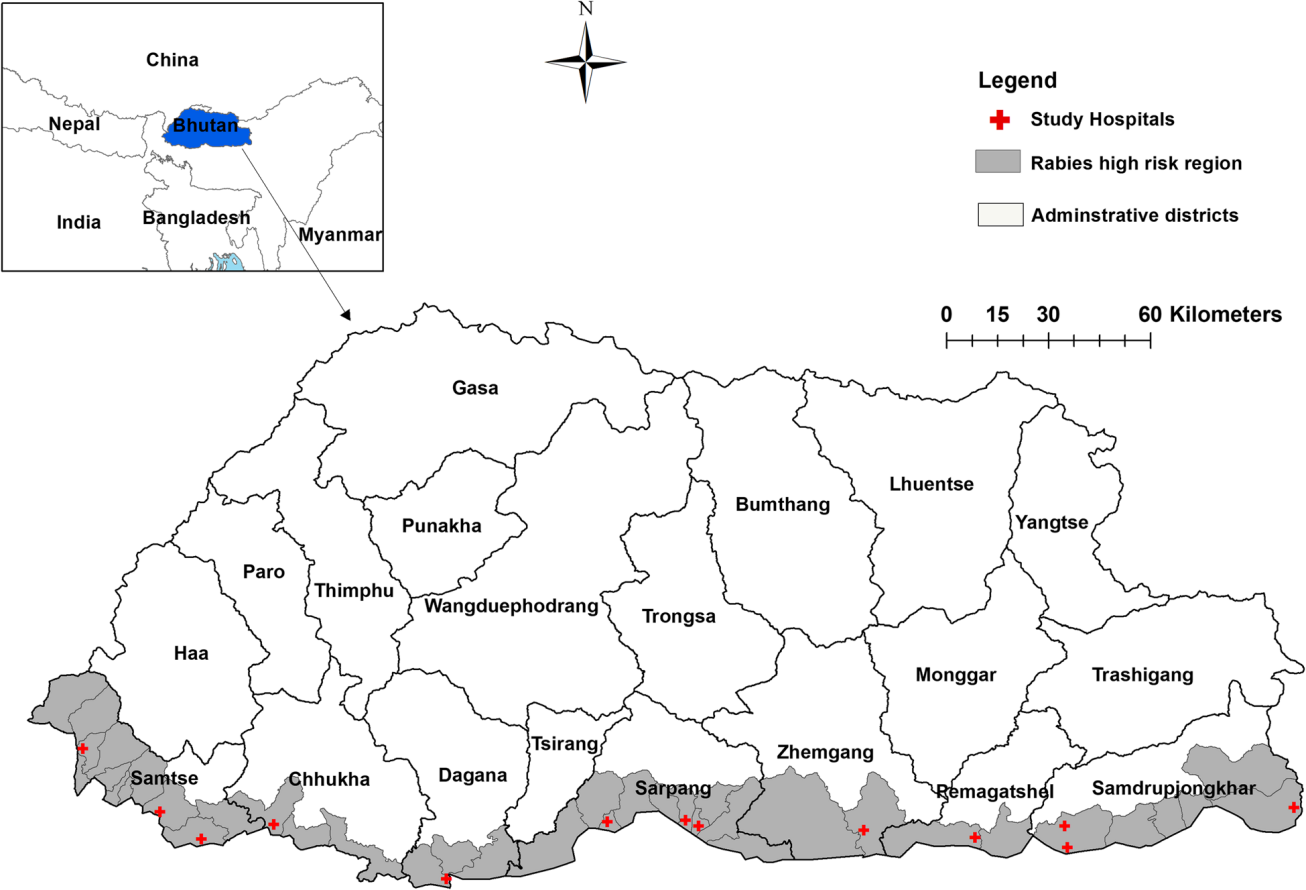
Map of Bhutan showing rabies high-risk regions and the location of the 13 study sites

All 13 health centers with doctors in the medical staff, i.e. hospitals and grade I Basic Health Units (BHU-I), located in the high rabies-risk belt of southern Bhutan were included in the study (Fig. 1). All clinicians involved in the management of human cases potentially exposed to rabies infection from animals or animal products in these health centers were included in the study. The term ‘clinician’ refers to staff who provide clinical management of cases in the health centers. This included doctors and paramedical staff (clinical officers and health assistants).

### Study design and data collection

Clinicians’ case management and PEP prescribing practices were evaluated by collecting information on the management of all people potentially exposed to rabies infection from animals or animal products who consulted with the clinicians during the period 1st February to 31st March 2016. The data collection period for this study was limited as it was part of a larger overarching programme to strengthen research capability of human health and animal health professionals in Bhutan and other South Asian countries. The case definition was thus any person presenting to a participating health center for clinical consultation to seek treatment for potential exposure to rabies from an animal or animal product. Hereafter, the term ‘case’ is used for those people who met the case definition and were included in the study.

Clinicians prospectively identified cases at the time they sought treatment at the health centers. Designated trained staff present in each study site completed a pre-tested questionnaire by observing each case’s consultation with the clinician. The questionnaire included demographic information about each case plus a set of 23 epidemiological questions to understand the nature of the animal exposure and to classify the risk of exposure to rabies into three categories - none, moderate, severe (see Additional file 1). These questions developed using the NRMG and a rabies expert panel, covered type of exposure, animal species involved, animal vaccination status (for dogs and cats) and potential rabies status of the animal (based on the typical clinical signs in animal like undue aggression, excessive salivation, indiscriminate biting of other animal or people and death of dog/cat within 10 days of observation period). The three possible types of rabies exposure were: direct exposure to an owned animal, direct exposure to a stray animal, and indirect exposure to any animal through contact with animal products or fomites. Investigators recorded which of the 23 questions were asked by the clinician and details of the PEP treatment prescribed. After the consultation, the investigator sought missing information and/or verification of the information collected during the consultation directly from the clinician or from the case as necessary. For example, details about the exposure event were collected from the case if the clinician did not elicit the required information during the consultation. Investigators recorded the clinicians’ rabies risk classification from the case sheet after the consultation. Where necessary the investigators validated the information collected during the consultation using copies of the PEP case record. Demographic information, level of qualification and years of experience were recorded for each clinician.

### Data analysis

#### Analysis of case data

Summary statistics were calculated to describe the demographics and exposure characteristics of the cases seeking treatment during the study period. Exact binomial tests were used to test for equi-probability of males versus females for various types of exposure.

#### Analysis of clinician data

Summary statistics were calculated to describe the demographics of the participating clinicians, the number of cases treated by each, and their rabies risk assessment and PEP treatment practices.

#### Evaluation of rabies risk assessment practices

The 23 epidemiological questions that were considered necessary to evaluate a case’s level of rabies risk were used to evaluate the clinicians’ risk assessment practices for each case, including the completeness of the questions asked by the clinician and the accuracy of their risk assessment. A particular subset of questions in the questionnaire was used to assess the rabies risk for each case depending on the type of exposure. For example, questions about indirect exposure were irrelevant for direct exposure cases. Similarly, questions about vaccination status of the animal were irrelevant for cases bitten by a stray animal.

Firstly, we described the proportion of relevant epidemiological questions pertaining to the relevant type of exposure that the clinician asked for each case. Secondly, we independently classified the rabies risk for each case into one of three categories (none, moderate, severe) by comparing the epidemiological information provided by the case, either during or following the consultation, with the risk criteria in the current NRMG [12]. The criteria that we used for rabies risk classification are presented in Table 1.

**Table 1:** Criteria for rabies risk assessment and recommended PEP prescription in case of potential exposure to “suspect or rabid animals”, extracted from the WHO adapted National Rabies Management Guidelines (2014) in Bhutan

Exposure type	Risk category	Recommended PEP
Licks on intact skin, touching, feeding of animals. ^1^Consumption of butter, curd, cheese, whey (dachu), cooked meat. Petting, bathing or coming in contact with utensils used on a suspected rabid animal.	None (Category 1)	Not recommended, if reliable case history available.
Person consuming unboiled or unpasteurized milk, buttermilk, uncooked meat from rabid animal. Nibbling of uncovered skin by potentially rabid animal. Minor scratches or abrasions without bleeding. Person who handles or prepares meat or handles carcass of rabid animals.	Moderate (Category 2)	Wound management, as appropriate Provide anti-rabies vaccine immediately Stop vaccination if animal remains healthy throughout the observation period of 10 days or if the animal is proven to be negative for rabies by a reliable laboratory using an appropriate diagnostic technique
Single or multiple transdermal bites or scratches Licks on broken skin Contamination with mucous membrane with saliva (i.e. licks or splash on oral cavity, eyes, nose, external genitalia)	Severe (Category 3)	Wound management Provide anti-rabies vaccine immediately Provide rabies immunoglobulin Stop vaccination if animal remains healthy as described above in Category 2

We described the proportion of cases for which the clinician had recorded a rabies risk classification in the case sheet. For cases for which the clinician had recorded a risk classification, we estimated the agreement between the risk category assigned by the clinician and the independently assessed risk category using the weighted Cohen’s kappa statistic for ordinal variables and equal weights. Thirdly, we performed logistic regression to identify factors associated with “agreement” between the clinician and the independent risk assessment, based on a binary outcome variable defined as: 0: disagreement between clinician and the NRMG, 1: agreement between clinician and the NRMG.. Variables evaluated in the bivariate model were:

Clinician variables:

- gender;
- professional experience (number of years);
- designation of the clinician: medical doctor, assistant clinical officer, health assistant;
- highest level of qualification: Bachelor of Medicine, Bachelor of Surgery (MBBS), Diploma or Certificate;

Health center variables

- health center type: Basic Health Unit, district hospital, regional referral hospital;

Risk classification

- actual level of risk of the exposure event according to the NRMG (none, moderate, severe).

Variables significant at *P* < 0.3 were used to fit a multivariate model. Observations were clustered by clinician, hospital and district, hence these 3 variables were used as nested random effects in the model. We used a stepwise backward model selection process using the lowest Akaike Information Criterion (AIC). Potential interactions between fixed effect variables in the final model were evaluated and selected using the lowest AIC.

#### Evaluation of PEP prescription practices

We described the proportion of cases for which clinicians prescribed PEP, in particular ARV and RIG, according to the clinicians’ rabies risk classification and the proportion prescribed according to our independent risk classification. All analyses were performed using R, in particular the mixed model was fitted using the package lme4 [13].

## Results

Fifty clinicians from the 13 health centers in southern Bhutan participated in this study. Data was collected for all 273 cases who sought treatment at the health care centers during the two-month study period. Each case had only one consultation for potential rabies exposure during the study period. Each clinician saw an average of 5.5 cases (median: four cases, range 1–19). Most consultations occurred in district level hospitals (177/273, 65%) followed by 22% in BHU-I and 14% in regional hospitals.

### Clinician demographics

The fifty clinicians comprised 21 medical doctors (MBBS), 5 clinical officers and 24 health assistants. Doctors had a median age of 28 years and a median of 2 years’ experience. Clinical officers and health assistants had a median age of 47 years and 42 respectively and a median of 21 and 19 years’ clinical experience. All clinical officers held a diploma while 10% of health assistants held a diploma and the remainder a certificate. Clinical officers and health assistants conducted the majority of consultations (55%) while doctors conducted the rest (45%) (Table 2).

**Table 2:** Demographic features of clinicians who led consultations for 273 cases potentially exposed to rabies in high risk areas in southern Bhutan during the period 1st February to 31st March 2016

Variable	Medical doctor	Clinical officer	Health assistant	Total
n (%)	21 (42%)	5 (10%)	24 (48%)	50
No. of cases seen (%)	124 (45%)	34 (13%)	115 (42%)	273
Age (median in years)	28	47	42	
Clinical experience (median in years)	2	21	19	
Gender				
Female (%)	4 (19%)	0 (0%)	8 (33%)	12 (24%)
Male (%)	17 (81%)	5 (100%)	16 (67%)	38 (76%)
Type of health center				
Basic Health Unit (%)	6 (29%)	0 (0%)	10 (42%)	16 (32%)
District Hospital (%)	12 (57%)	3 (60%)	8 (33%)	23 (46%)
Regional Hospital (%)	3 (14%)	2 (40%)	6 (25%)	11 (22%)
Highest qualification				
MBBS (%)	21 (100%)	0 (0%)	0 (0%)	21 (42%)
Diploma (%)	0 (0%)	5 (100%)	2 (8%)	7 (14%)
Certificate (%)	0 (0%)	0 (0%)	22 (92%)	22 (44%)

### Case demographics

**Fig. 2:**
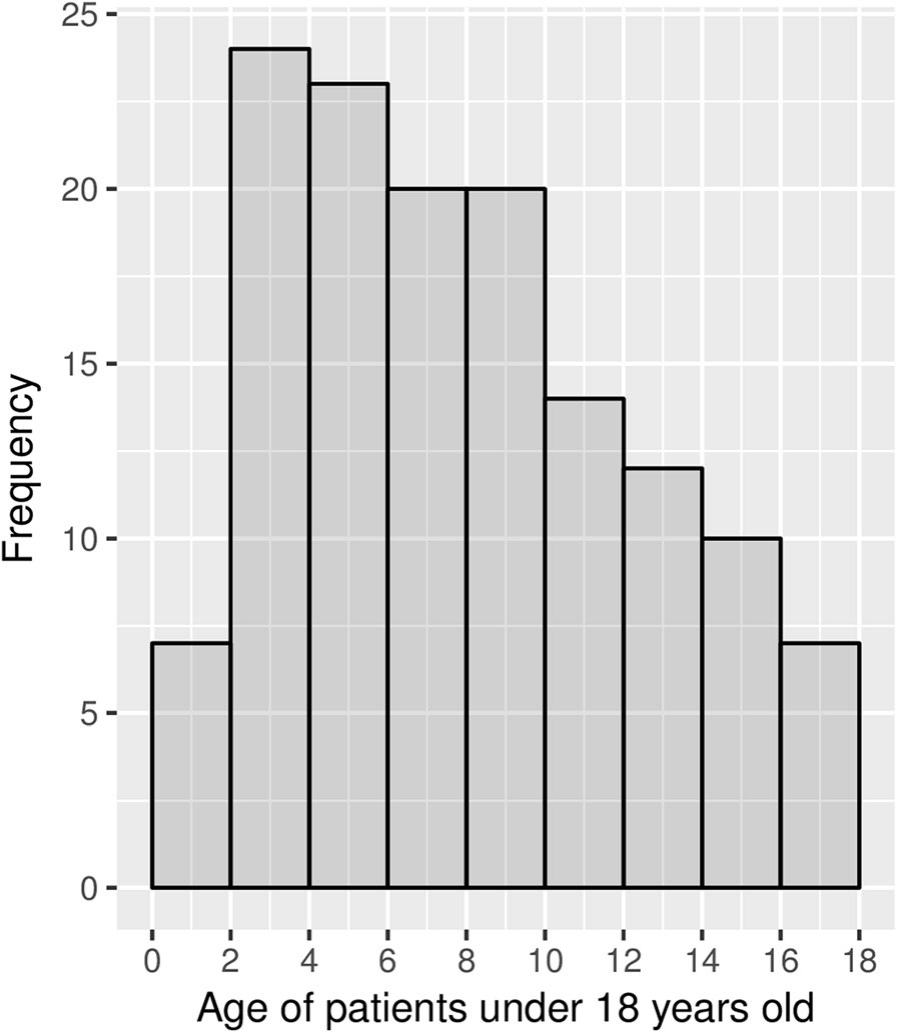
Age distribution of patients under 18 years old (*n* = 137) seeking treatment for potential exposure to rabies in the study centers during the period 1st February to 31st March 2016

**Fig. 3:**
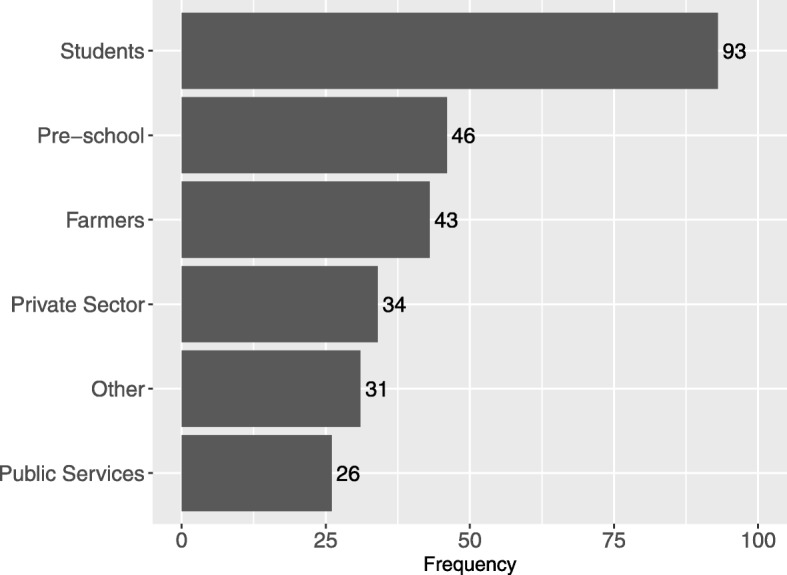
Occupation of 273 patients presenting for treatment following potential exposure to rabies in high-risk areas in southern Bhutan

The age of cases ranged from 2 to 85 years, with half under 18 years (137/273). The highest frequency of cases was 2 years old, following which the frequency decreased regularly with age (Fig. 2). Of the total cases, 97% were Bhutanese nationals; 55% were male, and 45% were female (*P* > 0.05). Half of the cases were preschoolers or students, and 16% were farmers (Fig. 3). The majority of cases (208/267, 78%) presented to the health center on the day of exposure or the following day. The animal species and types of exposure for the 273 cases are presented in Table 3. There was no significant difference between the proportion of male or female cases for each of the exposure categories (Table 3). The most common combination of species and exposure type was dog bites (189/273, 69%), including 67 (35%) inflicted by free-roaming (also referred to as ‘stray’) dogs and 123 (65%) by pet dogs. Eight cases reported that they were exposed through handling the carcass and/or drinking the milk from cattle or buffalo that were laboratory-confirmed cases of rabies. A further 8 cases were exposed to animals reported as showing typical clinical signs of rabies that had not been confirmed by laboratory diagnosis, including 6 cases exposed to dogs (5 dog bites), 1 to a cat and 1 to cattle/buffalo. In Bhutan, the confirmatory test for laboratory diagnosis of rabies used is Fluorescent Antibody Test (FAT) for antigen detection using fresh brain smear of animal.

**Table 3:** Animal species involved, type of exposure and demographics for 273 cases potentially exposed to rabies through contact with animals (categories not mutually exclusive)

Variable	Frequency (%)	Case demographics		
		Male	Female	Median age
Animal species (all exposure types)				
Pet dog	140 (51%)	79	61	18.5
Free roaming dog	81 (30%)	48	33	13.0
Cat	40 (15%)	16	24	20.0
Cattle/buffalo	12 (4%)	5	7	33.5
Rodents/wild animals	5 (2%)	2	3	27
Exposure types (all species)				
Bites (with bleeding)	152 (56%)	85	67	18.5
Bites (no bleeding)	61 (22%)	35	26	16.0
Scratches	52 (19%)	26	26	15.5
Licks or Nibbles	11 (4%)	5	6	22.0
Carcass handling (cattle/buffalo)	6 (2%)	3	3	39.0
Indirect exposure (consumption of milk/milk products or contact with animal products)	8 (3%)	3	5	28.0

### Rabies risk assessments

**Fig. 4:**
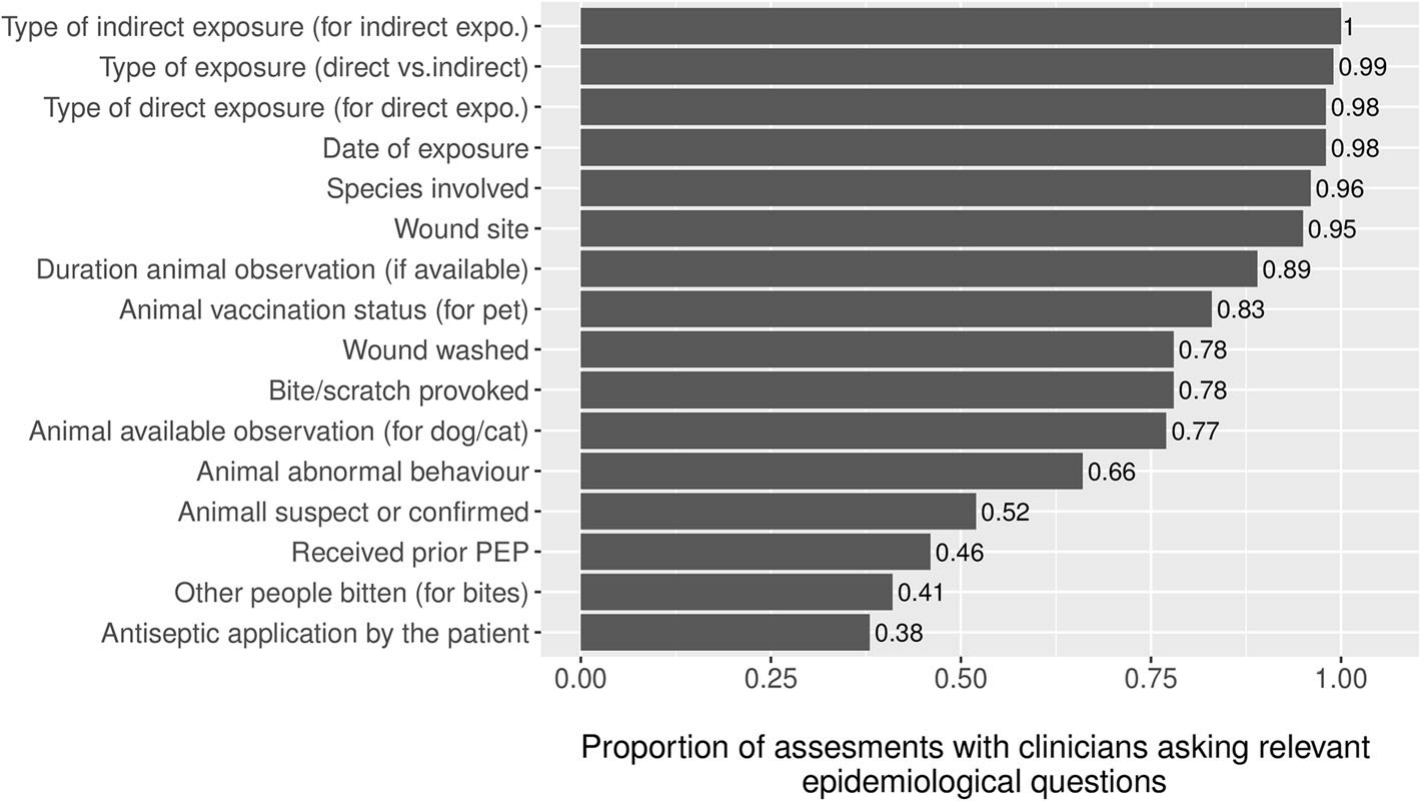
Proportion of relevant epidemiological questions asked by the clinicians for each exposure type (indicated in brackets); the denominator varied between 8 and 273, depending on the frequency of the type of exposure

In nearly all consultations, the clinician asked relevant questions about the type of rabies exposure. The type of exposure, the date, the wound site and the species involved were obtained in over 95% of consultations. Details of the epidemiological information that clinicians collected to assess rabies risk are detailed in (Fig. 4). The risk category for the 272 cases for whom information was available to independently classify rabies risk according to the NRMG was 57% severe risk, 43% moderate risk and only 1 (0.3%) was none. In one case the epidemiological information was missing. Clinicians recorded the rabies risk category (none, moderate, severe) on the case sheet for only 194 (71%) of the 273 cases. Of these 194 clinician risk assessments, only 102 (53%) were correctly classified when compared to the independent risk assessment (Table 4).

**Table 4:** Rabies risk classification stratified by clinician designation for 194 cases potentially exposed to rabies for which the clinician recorded a risk classification in 13 health centers in high rabies risk areas of southern Bhutan

Cases for which clinicians recorded the risk category (*n* = 194)					Cases for which clinicians did not record the risk category (*n* = 79)				
Clinician designation:	C^**1**^ (%)	U^**1**^ (%)	O^**1**^ (%)	Tot.	None^**2**^	Mod.^**2**^	Severe^**2**^	NA^**3**^	Percentage not recorded
Medical doctor	52 (52%)	40 (40%)	8 (8%)	**100**	0	12	12	0	**19.4%**
Clinical officer	9 (47%)	3 (16%)	7 (37%)	**19**	0	8	7	0	**44.1%**
Health assistant	41 (55%)	28 (37%)	6 (8%)	**75**	0	20	19	1	**34.8%**
Total	102 (53%)	71 (37%)	21 (11%)	**194**	0	40	38	1	**28.9%**

^1^: C = correct risk assessment, U = under-estimation of the risk, O = over-estimation of the risk

^2^: risk determined as per independent assessment using NRGM

^3^: impossible to determine the risk based on the questionnaire information

It was more frequent for clinicians to underestimate the rabies risk. Of the 116 cases independently classified as severe rabies risk for which the clinician recorded a risk, 62 (32%) were correctly classified by clinicians, 45 (23%) were misclassified as moderate risk, 9 (5%) were misclassified as having no rabies risk. The risk was not recorded for 38 (25%) of the cases independently classified as high risk. Based on results in Table 5, the kappa agreement test statistic was 0.203 (*p* < 0.001), indicating a poor agreement of rabies risk categorization between the clinicians and the independent risk categorization.

**Table 5:** Comparison of clinicians’ classification of rabies risk versus an independent classification of risk according to criteria listed in the NRMG (*n* = 273)

	Risk category assigned by the clinician				
	None	Moderate	Severe	NA^**2**^	Total
Independent risk category as per NRMG:					
None	0	1 (100%)	0	0	1
Moderate	17 (15%)	40 (34%)	20 (17%)	40 (34%)	117
Severe	9 (6%)	45 (29%)	62 (40%)	38 (25%)	154
NA^1^	0	0	0	1 (100%)	1
Total	26	86	82	79	273

^1^ One exposure event could not be categorized, even retrospectively, due to missing data in the questionnaire

^2^ Risk not recorded by the clinician

Potential explanatory variables associated with the agreement between the clinician’s risk assessment and the independent assessment (final model) are presented in Table 6. The effect of clinical designation interacted with that of gender. Male health assistants were the most likely to make an accurate risk assessment and female health assistants were the least likely. Female and male doctors were not significantly different. Male health assistants were three times more likely to make an accurate risk assessment than doctors, whereas doctors were twice as likely to make an accurate assessment compared with female health assistants. Male health assistants were 12 times more likely to make an accurate risk assessment than female health assistants. Clinicians from regional or district hospitals were significantly more likely to conduct accurate risk assessments compared to clinicians in Basic Health Units (Odds Ratio of 17.6 and 7.8, respectively), independent of the clinician’s designation in the different health centers. The random effects (clinicians nested in hospitals nested in districts) suggested that after taking into account the variation in assessment accuracy associated with clinicians, hospitals and the fixed effects, there was no residual variation between districts.

**Table 6:** Final multivariate logistic regression model of factors associated with clinicians making an accurate rabies exposure risk assessment, defined by agreement of clinicians’ risk assessment with an independent assessment based on criteria listed in the NRMG

Variable	Coefficient Estimate	SE	Multiplier^1,3^	*P*-value
Gender				
Male				
Female	−0.77	0.55	0.5	0.16
Designation				
Medical Doctor				
Clinical Officer	−0.70	0.63	0.5	0.26
Health Assistant	1.10	0.49	3.0	0.02
Health Centre Type				
Basic Health Unit				
District Hospital	2.05	0.73	7.8	0.00
Regional Hospital	2.87	1.16	17.6	0.01
Female*Health Assistants^2^	−1.70	0.84	0.2	0.04

^1^ Multiplicative effect on the odds of the outcome, due to being in this stratum compared to the reference stratum. This corresponds to the odds ratio only for variables for which there were no interactions in the model

^2^The interaction between Gender and Designation; there were no female Clinical Officers in the study

^3^The Odds Ratio for varying levels of gender and designations can be obtained by combining the corresponding coefficients, e.g. OR of correct risk assessment for male health assistant compared to female health assistants is exp.(1.10)/exp.(− 0.77 + 1.10–1.70) = 12.

### Clinicians’ PEP prescription practices

The number of cases for whom clinicians prescribed ARV by rabies risk category as assessed by the clinician and as independently assessed according to criteria in the NRMG is shown in Table 7. There were 271 patients eligible to receive ARV according to the independent risk classification (moderate or severe risk). Among these, clinicians prescribed ARV for 92% (248/271) including 75 cases for whom a risk assessment was not recorded. Neither ARV nor RIG was prescribed for 1 case assessed by the clinician to be severe risk and ARV was not prescribed for another 2 cases assessed by the clinicians to be moderate risk. Conversely, clinicians prescribed ARV for 10 (38%) of 26 cases whom they assessed as having no rabies risk and 75 (95%) of 79 patients for whom they did not record a rabies risk on the case sheet. Considering the independent risk categorization of cases, 7 (5%) of 154 severe risk and 16 (14%) of 117 moderate risk cases were not prescribed ARV. RIG was prescribed in addition to ARV for three cases (Table 7). One case had handled the carcass of a cow confirmed with rabies by laboratory tests and was misclassified as severe risk, and two cases had been bitten by a dog clinically suspected of being infected with rabies, one of which was misclassified as moderate risk and the other correctly classified as severe risk.

**Table 7:** Number of cases prescribed ARV or RIG by rabies risk category as assessed by clinicians and as independently assessed according to criteria in the NRMG

	Clinician risk classification				Independent risk classification (NRMG)				Total
ARV prescription:	None	Mod.	Severe	NA	None	Mod.	Severe	NA	
No ARV	16	2	1	4	0	16	7	0	23
ARV	10	84	81	75	1	101	147	1	250
RIG	0	1	2	0	0	1	2	0	3
Total^1^	26	86	82	79	1	117	154	1	273

^1^ Categories are not mutually exclusive: the 3 patients receiving RIG also received ARV

NA = not assessed; ARV: anti-rabies vaccine; RIG: Rabies immunoglobulin.

## Discussion

This is the first study to evaluate clinicians’ management and PEP practices for human cases potentially exposed to rabies in Bhutan. All 50 clinicians working in the 13 health centers in the high rabies risk areas of southern Bhutan were evaluated in the study, including 2 regional hospitals, 6 district hospitals and 2 BHU-Is. Amongst the 50 clinicians, 42% (21/50) were medical doctors with a MBBS degree, 10% were clinical officers who held a diploma and 48% were health assistants of whom 8% held a diploma and 92% held a certificate (Table 2). Doctors had a median of only two years of experience, while clinical officers and heath assistants had a median of 21 and 19 years’ experience respectively. Each clinician conducted a median of four consultations (range 1–19). Doctors and health consultants conducted a similar proportion of consultations, 45 and 42% respectively, while clinical officers conducted only 13%. Clinical officers practiced only in BHU while health assistants and doctors practiced both in BHU and hospitals.

Guidelines for management of rabies cases is provided in the NRMG (2014). This guideline is based on WHO recommendations. The NRMG recommend a rabies risk assessment is conducted for all cases exposed to “suspected or confirmed rabid animals” by collecting epidemiological information on the exposure history of the case and provides guidance for PEP for each risk category. The NRMG provides guidelines for classifying rabies risk into three categories: none, moderate or severe risk, and recommends prescription of ARV for those in the moderate and severe categories. Additional RIG is recommended for cases in the severe risk category. Where possible, we assessed the accuracy of clinicians’ risk assessments by comparing these with an independently classified risk assessment using the NRMG guidelines, based on the patient’s interview. We also evaluated the appropriateness of the PEP prescription by comparing the clinicians’ prescription against their own risk assessment and against the independent risk assessment. Clinicians recorded the rabies risk classification for only 71% of cases. However, they did collect some information on the type of exposure for nearly all cases, which could have contributed to risk categorization. Clinical officers did not record the risk category for 44% of the cases compared with 35% for health assistants and 19% for doctors (Table 4). It is not known if clinicians did not record the risk category because they did not make a decision about the risk category or if they did make a decision but did not record this. There is no clear definition for a “suspected” animal rabies case in the NRMG, which may contribute to clinicians not categorizing the rabies risk of a case. Since rabies is endemic in southern Bhutan, all animals involved in an exposure event should be suspected of being infected with rabies, with appropriate risk assessment performed and documented and PEP prescribed accordingly.

The clinicians’ rabies risk categorization showed a low level of agreement with the independent assessment using the NRMG (kappa = 0.203). Of the 194 cases for which clinicians recorded a rabies risk category, only 53% were correctly classified. Nearly all rabies risk assessments took into account the type of exposure (Fig. 4), the latter being clearly outlined in the national guidelines (Table 1). However, they often omitted or ignored relevant epidemiologic information necessary to classify the risk appropriately. As a result, clinicians tended to underestimate the exposure risk. It is a concern that clinicians mis-classified 13% of cases as having no risk, while they were independently assessed as having a moderate or severe risk. Overall, male health assistants were the most likely group of clinicians to make an accurate risk assessment, while female health assistants were least likely (Table 6). Surprisingly, doctors did not perform as well as male health assistants, similar to findings from an Indian study [14]. Clinicians from district or regional hospitals were more likely to perform better than clinicians in BHUs (Odds Ratios of 7.8 and 17.6, respectively) regardless of clinician type. These results possibly reflect greater opportunities for clinicians in hospitals to participate in capacity building training programs for rabies conducted by the Ministry of Health, as observed in another study in Haiti [15]. The attitude of clinicians towards training might also differ depending on their level of qualification. Health assistants are more readily available to attend such trainings. Most doctors, however, are unable to attend or oblivious to training sessions, possibly due to high workloads. The apparently better performance of male health assistants compared to male doctors may reflect the impact of such continuing education opportunities, rather than initial education level. The poorer performance of female health assistants compared to male health assistants could arise from fewer opportunities to participate in continuing education training by female staff. Additionally, male health assistants had significantly more work experience (median of 29 years) compared with male and female doctors (median of 2 years) and female health assistants (median of 10 years). The poorer performance of clinicians in BHUs compared to hospitals, independent of clinician type, might be associated with less training and poorer awareness of the NRMG guidelines amongst BHU-level staff. This might also partially reflect clinicians in BHUs having fewer years of clinical experience, even though the coefficients for type of health center were virtually unchanged when adding this variable to the model, after accounting for type of clinician. Nevertheless, junior clinicians are often posted to lower level health facilities rather than hospitals, as per government policies. Given the majority of rabies assessments (78%) occurred in hospitals rather than BHUs, there may be less opportunity for junior clinicians in BHUs to gain experience if this pattern represents the general pattern of consultations.

The inaccuracy of clinicians’ risk assessments was compensated for by the prescription of ARV for the majority of cases (91.6%, Table 7). The rabies risk in all cases but one was independently classified as moderate or severe, hence ARV was prescribed for most cases who needed this, based on their independent risk classification. However, clinicians prescribed ARV for 10 of 26 cases whom they classified as having no rabies risk. These results are reflective of findings of a nationwide study conducted between 2005 and 2008, which reported frequent PEP administration in category I exposures [10]. The clinicians may be erring on the safe side with respect to administering ARV; they may also be under pressure from their patients to prescribe ARV. Alternatively, some clinicians may just be using ARV in the absence of conducting a rabies risk assessment. Such practices are likely to result in unnecessary costs associated with implementation of PEP to prevent human cases of rabies. On the other hand, clinicians did not prescribe ARV for 2.4% of cases whom they had classified as moderate risk and 1.2% of cases classified as severe risk. This could potentially represent a public health threat.

In this study, 8 cases were exposed to laboratory-confirmed rabid cattle or buffaloes through handling the carcasses and/or drinking milk from these animals. This exposure is considered a moderate exposure risk in the NRMG, requiring ARV treatment but not RIG. One of these 8 cases was incorrectly classified by the clinician as having no risk and the case did not receive the required ARV. Another two of these cases were incorrectly classified as severe risk. Both received ARV, and one also received RIG, which is not consistent with the NRMG. An additional 8 cases were exposed to animals reported as showing typical clinical signs of rabies that had not been confirmed by laboratory diagnosis; included 6 cases exposed to dogs (5 dog bites), 1 to a cat and 1 to cattle/buffalo. All 5 cases that were bitten by dogs exhibiting pathognomonic rabies symptoms received ARV, but no RIG. One of these cases should have been categorized as severe risk, given the presence of puncture wounds, and should have received RIG. Another 76 cases were exposed to animals that clinicians classified as “suspected of rabies” including 62 dog bites and 47 with bleeding wounds. According to the NRMG these cases should have been classified as severe risk and should have been prescribed RIG. Overall, RIG was prescribed to only 3 cases (1%), one exposed to a laboratory-confirmed rabid cow and the other 2 bitten by dogs for which no pathognomonic signs of rabies were reported. This concurs with the results of the earlier study that RIG was not regularly administered to dog bite victims in Bhutan [10]. While WHO recommends RIG is prescribed for patients bitten by suspected rabid animals, the availability of expensive RIG is very limited in Bhutan, as is true in many rabies endemic countries [16]. These results indicate the need for clearer guidance in the NRMG regarding prescription of RIG. Given the limited availability of RIG in Bhutan it could be valuable to define criteria for an “extra-severe” risk category to prioritize for RIG prescription. Another area of the NRMG that is not clearly understood by clinicians is the criteria for risk categorization associated with exposure to dairy products and meat consumption. Criteria for risk categorization of these exposures are not clearly outlined in the WHO guidelines, and were added to the NRMG due to the high prevalence of animal product consumption during animal rabies outbreaks in Bhutan. The above results indicate that there is a need to improve the clarity of guidelines in some areas of the NRMG and to train clinicians in the interpretation of these guidelines.

This study was limited by a small sample size and the limited 2-month study-period in late winter and early spring. Previous studies show that PEP prescriptions follow a seasonal pattern, with an increase in winter and spring [10]; a very similar pattern is also observed in dog bites in Bhutan [9]. Therefore, this study might not be representative of year-round case demographics. However, seasonality is unlikely to affect clinicians’ rabies risk assessment. Prior communication with clinicians requested them to manage cases as they would normally. Observational biases associated with the interviewers were mitigated by providing comprehensive training on information recording. However, clinicians may have been influenced towards conducting more rigorous risk assessments during the study. Despite these limitations, the results of the study are consistent with those of a previous study in Bhutan [10].

Good clinical judgment is essential to prevent human rabies [17]. Other studies on clinician’s knowledge and attitudes conducted in the USA showed an unsatisfactorily low level of compliance with national guidelines resulting in inappropriate PEP treatment [18–20]. Greater compliance with guidelines is important to achieve more cost-effective PEP use [21, 22]. For example, a study in a low rabies-risk area in Massachusetts, USA highlighted how large amounts of rabies PEP could be wasted in patients with low or non-existent risk of rabies exposure [23]. Studies in countries free of rabies [24] and in endemic areas [25–27] similarly reported insufficient rabies risk assessment due to clinicians’ lack of familiarity with the recommendations, highlighting the need to update clinicians’ knowledge [14]. Discordant rabies risk assessment and PEP practice was also apparent in our study. In contrast, public health physicians in Israel showed a very high level of compliance with PEP guidelines [28]. This could be due to different public health policies, better training or expertise of clinicians undertaking rabies assessments and better access to immunoglobulin in Israel compared with Bhutan.

This study highlighted important gaps in clinicians’ management of human cases potentially exposed to rabies in high rabies risk areas of Bhutan. The cost-effectiveness of applying PEP to prevent rabies in these areas could be improved by reviewing and updating the NRMG and providing more training for clinicians in using the NRMG to manage cases potentially exposed to rabies. Our findings indicate that rabies training should target medical doctors, female health assistants and clinicians in BHUs and take into account the availability and the motivation of clinicians. In addition, the WHO recommends a One Health approach and the use of Integrated Bite Case Management integrating animal health and public health sectors [4]. Better public health outcomes and concurrent PEP savings could be achieved by systematic notification to the animal health sector of human cases that have been exposed to suspected rabid animals. In turn, appropriate quarantine and surveillance of the animals involved can be conducted. If the animal is confirmed free of rabies there is no need for further administration of ARV for the exposed case(s), which can reduce the expenditure associated with PEP.

Based on the results of this study, there is scope for significant improvements of national rabies management policies and clinician training in Bhutan. This may contribute to reaching the national goal of eliminating dog-mediated human rabies by 2030.

## Supplementary information

**Additional file 1.** Study Questionnaire.

## Data Availability

The data is available from the corresponding author on request.

## Abbreviations

AIC: Akaike Information Criterion
ARV: Anti-Rabies Vaccine
BHU: Basic Health Units
CNR: College of Natural Resources, Royal University of Bhutan
FAT: Fluorescent Antibody Test
FNPH: Faculty of Nursing and Public Health, Khesar Gyalpo University of Medical Sciences of Bhutan
MBBS: Bachelor of Medicine, Bachelor of Surgery
NRMG: National Rabies Management Guidelines
PEP: Post Exposure Prophylaxis
REBH: Research Ethics Board of Health
RIG: Rabies Immunoglobulin
USA: United States of America
USD: US Dollar
WHO: World Health Organization
