# Supplementary material for: Evaluation of post-exposure prophylaxis practices to improve the cost-effectiveness of rabies control in human cases potentially exposed to rabies in southern Bhutan
Source: BMC Infect Dis. 2020 Mar 6;20:203. doi: 10.1186/s12879-020-4926-y (PMC7060656; doi:10.1186/s12879-020-4926-y)
Supplement: Supplementary file 1 — Additional file 1. Study Questionnaire. [file 12879_2020_4926_MOESM1_ESM.doc]

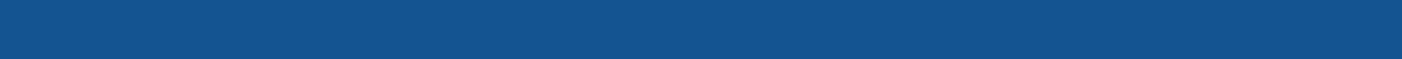
One Health Epidemiology Fellowship Program


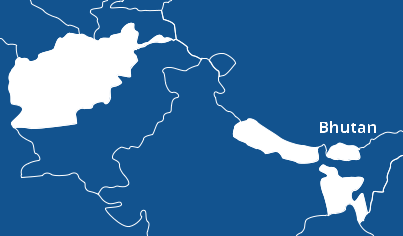


Af ghanist an

Nepal

Bangladesh

Integrating Education and Action for One Health

Afghanistan ı Bangladesh ı Bhutan ı Nepal

Protecting and improving the health of people, animals & wildlife

**Assessment of knowledge, attitude and practices of clinicians related to rabies post-exposure treatment in high-risk areas of Bhutan**

**QUESTIONNAIRE-B: TO BE COMPLETED BY THE DESIGNATED HEALTH STAFF**

**(2016)**

**General Instruction:**

- This questionnaire consists of section A, B, C, D and E.
- This Questionnaire shall be completed by a designated health staff who is trained by the Principal Investigators (One Health Fellows).
- The designated health staff will complete this Questionnaire in the selected Clinician’s chamber as and when the Clinician treat a patient that fulfil the Case definition.
- **A Case Definition:**

For this study a **Case** is defined as a patient reporting to the health centre with **Direct Exposure** (such as a person bitten, scratched, nibbled or licked by any type of animals including wild animals, or a person handling carcass of suspected or rabid animals) or **Indirect Exposure** (such as consumption of animal products like meat, milk, dairy products of a suspected or rabid animal) to an animal in the high-risk rabies endemic area.

- For this study a ‘Rabies High-risk Area’ is defined as those *geogs* (administrative block) in southern Bhutan bordering India or that had reported at least one outbreak per year in animals for ≥3 years in the past 10 years.
- The quality of information recorded in terms of completeness, correctness and timeliness will be ensured by the designated health staff. In addition, it shall be monitored and assisted by the Principal Investigators (One Health Fellows).
- A designated staff shall also maintain a photocopy of a patient’s case sheet with all the required information at each health facility for the reference.
- The Principal Investigators shall train at least two health staff from each selected health centre on the study objectives and how to complete this questionnaire prior to the start of the study.
- The study will be implemented with effect from 1^st^ February to 31^st^ March 2016.
- In addition to this Questionnaire-B, a one-time self-administered knowledge, attitude and practices (KAP) Questionnaire on Rabies and its PEP will be administered to the clinicians in the presence of the Principal Investigators in the selected study sites.

For additional information or inquiry about the study or questionnaire, please contact the following investigators at the given contact details:

1. Dr. Kinley Penjor, MPH Fellow; Mobile no. +975 17619191 Email id:Kinlaypal@gmail.com
2. Dr. Chendu Dorji, MVM Fellow; Mobile no. +975 16936309 Email id: dorjivet@yahoo.com
3. Dr. Kinley Penjor, MVM Fellow; Mobile no. +975 16900620 Email id: kinleypenjor26@yahoo.com

**Funded by:** **Implemented by:** **In collaboration with:**

**
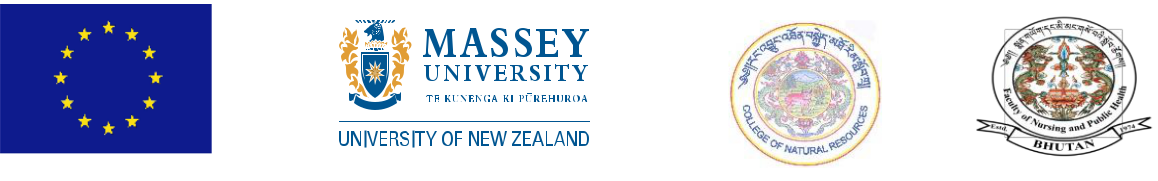
**

**Assessment of knowledge, attitude and practices of clinicians related to rabies post-exposure treatment in high risk areas of Bhutan**

Questionnaire No.

(3-digit letter for the health centre

followed by 3-digit number for a Case

patient)

Date of Recording:

DD/MM/YY

Recorded by (Name of designated health

staff):

**A. Health Centre Information**

| A01. Name of Health Centre: |  |  |  |  |  |
| --- | --- | --- | --- | --- | --- |
|  |  |  |  |  |  |
| A02. Location of Health Centre: |  |  |  |  |  |
|  |  |  |  |  |  |
| A03. Geog: |  |  |  |  |  |
|  |  |  |  |  |  |
| A04. District: |  |  |  |  |  |
|  |  |  |  |  |  |
| A05. Type of Health Centre. |  |  | ✓ | | |
|  |  | (Please tick ( ) one: | | | |
|  |  | ☐ Regional Referral Hospital……………….…………………........1 | | | |
|  |  | ☐ District Hospital……………………………….……………..…........2 | | | |
|  |  | ☐ Basic Health Unit-I (BHU-I)……………………….……………….3 | | | |
|  |  |  |  |  |  |
| **B. Details of the Patient** |  |  |  |  |  |
| B01. Patient Name: |  |  |  |  |  |
|  |  |  | | |  |
| B02. Age: |  | Age rounded to years: | | |  |
|  |  |  |  |  |  |
|  |  |  | | |  |
| B03. Gender: |  | Please tick ( ^✓^) one: | | |  |
|  |  | ☐ Female……………………………………………………..…………….…1 | | |  |
|  |  | ☐ Male……………………………………………………..………………....2 | | |  |
|  |  |  |  |  |  |
|  |  |  |  | **Page 3 of** 14 | |

| B04. Contact No: |  | Mobile/land line | | | | | | | | |
| --- | --- | --- | --- | --- | --- | --- | --- | --- | --- | --- |
|  |  |  |  |  |  |  |  |  |  |  |
|  |  |  |  |  |  |  |  |  |  |  |
| B05. Resident Place/Location: |  |  |  |  |  |  |  |  |  |  |
|  |  |  |  |  |  |  |  |  |  |  |
| B06. Geog: |  |  |  |  |  |  |  |  |  |  |
|  |  |  |  |  |  |  |  |  |  |  |
| B07. District: |  |  |  |  |  |  |  |  |  |  |
|  |  |  |  | | | | | | | |
| B09.Nationality: |  |  | ✓ | | | | | | | |
|  |  | Please tick ( ) one. | | | | | | | | |
|  |  | ☐ National ………………..………………………………..……………....1 | | | | | | | | |
|  |  | ☐ Foreigner……….……………………………………..….………...…...2 | | | | | | | | |
|  |  |  | | | | | | | | |
| B08. Occupation: |  | Please tick ( ^✓^) one. | | | | | | | | |
|  |  | ☐ Pre-school……………………………………………………..………....1 | | | | | | | | |
|  |  | ☐ Student……………………………….……………..……..……………..2 | | | | | | | | |
|  |  | ☐ Trainee………………………………………….………….……..……....3 | | | | | | | | |
|  |  | ☐ Civil servant………………………………………………..…………....4 | | | | | | | | |
|  |  | ☐ Private sectors….………………………………..….…………….…..5 | | | | | | | | |
|  |  | ☐ Construction worker …………….…………..………………….....6 | | | | | | | | |
|  |  | ☐ Military….……………………….…………….……….………....….….7 | | | | | | | | |
|  |  | ☐ Monks/Nuns………………………..……….…..….……………….…8 | | | | | | | | |
|  |  | ☐ Farmer……………………………….………..…….……..……….….…9 | | | | | | | | |
|  |  | ☐ Unemployed………………………………..….……………………..10 | | | | | | | | |
|  |  | ☐ Others (specify)……………………………………………………….11 | | | | | | | | |
|  |  | ………………………………………………………………………………… | | | | | | | | |
|  |  |  |  |  |  |  |  |  |  |  |

Page **4** of **14**

**C. Clinician’s details**

| C01. | Clinician’s Unique ID Code: |  |  |  |  |  |  |  |  |  |  |  |  |  |  |  |
| --- | --- | --- | --- | --- | --- | --- | --- | --- | --- | --- | --- | --- | --- | --- | --- | --- |
|  |  |  |  |  |  | | |  |  |  | |  |  |  |  |  |
| C02. | Age: |  | Age rounded to years: | | | | | | | | | | | | | |
|  | |  |  |  |  |  |  |  |  |  |  |  |  |  |  |  |
|  | |  |  |  |  |  |  |  |  |  | |  |  |  |  |  |
| C03. Gender: | |  | Please tick ( | | | | ✓ | | | | | | | | | |
|  |  |  |  |  |  |  |  | ) one: | | | | | | | | |
|  |  |  | ☐ Female…………………………………………………………………………1 | | | | | | | | | | | | | |
|  |  |  | ☐ Male………………………………………………………………..………....2 | | | | | | | | | | | | | |
|  | |  |  | |  | | | |  | | | | | | | |
| C04. Contact No: | |  | Mobile number: | | | | | | | | | | | | | |
|  |  |  |  |  |  |  |  |  |  |  | |  |  |  |  |  |
|  |  |  |  |  |  |  |  | |  | | | | | | | |
| C05. | Designation: |  | Please tick ( | | | | ✓ | | | | | | | | | |
|  |  |  |  |  |  |  |  | ) one: | | | | | | | | |
|  |  |  | ☐ Medical Officer…..……………………………………….…….…….....1 | | | | | | | | | | | | | |
|  |  |  | ☐ Specialist………….……………………………………….………….….….2 | | | | | | | | | | | | | |
|  |  |  | ☐ ACO/CO…..……………….……………………….…………..…………...3 | | | | | | | | | | | | | |
|  |  |  | ☐ HA…………………………………………………………….……….……..…4 | | | | | | | | | | | | | |
|  |  |  |  |  |  |  |  | |  | | | | | | | |
| C06. | Highest qualification obtained: |  | Please tick ( | | | | ✓ | | | | | | | | | |
|  |  |  |  |  |  |  |  | ) one: | | | | | | | | |
|  |  |  | ☐ Master……………………..………………………………..……..…………1 | | | | | | | | | | | | | |
|  |  |  | ☐ MBBS ………………..…………………………………….………………….2 | | | | | | | | | | | | | |
|  |  |  | ☐ Degree (other than MBBS)……………………….………………….3 | | | | | | | | | | | | | |
|  |  |  | ☐ Diploma ………………………………………………..……….…..……….4 | | | | | | | | | | | | | |
|  |  |  | ☐ Certificate…………………………………………….……….…………….5 | | | | | | | | | | | | | |
|  |  |  |  | | | | | | | | | | | | | |
| C07. | Number of years in |  | Please round it off to the nearest years: | | | | | | | | | | | | | |
|  | practice/experience: |  |  |  |  |  |  |  |  |  |  |  |  |  |  |  |
|  |  |  |  |  |  |  |  |  |  |  |  |  |  |  |  |  |
|  |  |  |  |  |  |  |  |  |  |  |  |  |  |  |  |  |

Page **5** of **14**

**D. Epidemiological information:**

| **No.** | **Question** | **Interviewer’s** | |  |  | **Patient’s response** | | | | | | | | **Go To** |
| --- | --- | --- | --- | --- | --- | --- | --- | --- | --- | --- | --- | --- | --- | --- |
|  |  | **Observation** | |  |  |  |  |  |  |  |  |  |  |  |
|  |  |  |  |  |  |  | | | | | | | |  |
| D01 | Did the clinician ask the | Please tick ( | ✓ | ) one: |  | Date of exposure in DD/MM/YY | | | | | | | |  |
|  | patient about the date |  |  |  |  |  |  |  |  |  |  |  |  |  |
|  |  | ☐ Yes |  |  |  |  |  |  |  |  |  |  |  |  |
|  | of animal exposure? |  |  |  |  |  |  |  |  |  |  |  |  |  |
|  |  | ☐ No |  |  |  |  |  |  |  |  |  |  |  |  |
|  |  |  |  |  |  |  |  |  |  |  |  |  |  |  |
|  |  |  |  |  |  |  |  |  |  | | | | |  |
| D02 | Did the clinician ask | Please tick ( | ✓ | ) one: |  | Please tick ( | | | ✓ | | | | |  |
|  | about the type of |  |  |  |  |  |  |  | ) one: | | | | |  |
|  |  | ☐ Yes |  |  | ☐ Direct animal exposure (e.g. bites, | | | | | | | | |  |
|  | animal exposure? |  |  |  |  |  |  |  |  |  |  |  |  |  |
|  |  | ☐ No |  |  |  | scratches, licks, nibbling, kissing, | | | | | | | |  |
|  |  |  |  |  |  | handling of carcass)…………………….1 | | | | | | | |  |
|  |  |  |  |  |  |  |  |  |  |  |  |  |  |  |
|  |  |  |  |  | ☐ Indirect animal exposure (e.g. | | | | | | | | | **D04** |
|  |  |  |  |  |  | consumption of dairy products)….2 | | | | | | | |  |
|  |  |  |  |  |  |  |  |  |  |  |  |  |  |  |
| D03 | Did the clinician ask | Please tick ( ^✓^) one: | | | Please tick ( | | | | ✓ | | | | |  |
|  | what the type of direct |  |  |  |  |  |  |  | ) one or more: | | | | |  |
|  |  | ☐ Yes |  |  | ☐ Bite wound with bleeding………..…..1 | | | | | | | | |  |
|  | animal exposure is? |  |  |  |  |  |  |  |  |  |  |  |  |  |
|  |  | ☐ No |  |  | ☐ Bite without bleeding…………….......2 | | | | | | | | |  |
|  |  |  |  |  | ☐ Scratches…..…………..………….………..3 | | | | | | | | |  |
|  |  |  |  |  | ☐ Licks on the mucus membrane | | | | | | | | |  |
|  |  |  |  |  |  | or broken skin………………………….....4 | | | | | | | |  |
|  |  |  |  |  | ☐ Licks on the intact skin…………….…..5 | | | | | | | | |  |
|  |  |  |  |  |  | ☐ Nibbling on mucus membrane or | | | | | | | |  |
|  |  |  |  |  |  | broken skin………………….................6 | | | | | | | |  |
|  |  |  |  |  | ☐ Nibbling on intact skin…………………7 | | | | | | | | |  |
|  |  |  |  |  | ☐ Handling carcass …………………………8 | | | | | | | | |  |
|  |  |  |  |  | ☐ Other direct exposure (specify)…..9 | | | | | | | | |  |
|  |  |  |  |  | ……………………………………………………….… | | | | | | | | |  |


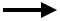


**Page 6 of** 14

| **No.** | **Question** | **Interviewer’s** | |  | **Patient’s response** | | **Go To** |
| --- | --- | --- | --- | --- | --- | --- | --- |
|  |  | **Observation** | |  |  |  |  |
| D04 | If the exposure is due to | Please tick ( | ✓ | ) one: | Please tick ( | ✓ |  |
|  | indirect exposure, did |  |  |  |  | ) one or more: |  |
|  |  | ☐ Yes |  |  | ☐ Consumption of raw milk.…………..1 | |  |
|  | the clinician ask about |  |  |  |  |  |  |
|  | the type of exposure? | ☐ No |  |  | ☐ Consumption of boiled milk………..2 | |  |
|  |  |  |  |  |  |  |  |
|  |  |  |  |  | ☐ Consumption of uncooked meat…3 | |  |
|  |  |  |  |  | ☐ Consumption of cooked meat…….4 | |  |
|  |  |  |  |  | ☐ Consumption of butter, buttermilk, | |  |
|  |  |  |  |  | cheese or curd, whey………..………5 | |  |
|  |  |  |  |  | ☐ Contact with utensils of animal on | |  |
|  |  |  |  |  | mucus membrane or broken | |  |
|  |  |  |  |  | skin……………………………………………6 | |  |
|  |  |  |  |  | ☐ Contact with utensils of animal on | |  |
|  |  |  |  |  | intact skin………….…......................7 | |  |
|  |  |  |  |  | ☐ Other indirect exposure (specify).8 | |  |
|  |  |  |  |  | ……………………………………...…….………..…. | |  |
| D05 | Did the clinicians ask | Please tick ( | ✓ | ) one: | Please tick ( | ✓ |  |
|  | about the animal |  |  |  |  | ) one or more: |  |
|  |  | ☐ Yes |  |  | ☐ Pet dog………………………...............…1 | |  |
|  | species responsible for |  |  |  |  |  |  |
|  | the exposure? | ☐ No |  |  | ☐ Pet cat…………………………….…….......2 | |  |
|  |  |  |  |  |  |  |  |
|  |  |  |  |  | ☐ Free-roaming dog……….............…..3 | |  |
|  |  |  |  |  | ☐ Free-roaming cat………………….…....4 | |  |
|  |  |  |  |  | ☐ Rat/rodent….………………………………5 | |  |
|  |  |  |  |  | ☐ Cattle/Buffalo…………...............……6 | |  |
|  |  |  |  |  | ☐ Sheep/Goat……….………………..…....7 | | **D08** |
|  |  |  |  |  | ☐ Pig…………………….……………….……...8 | |  |
|  |  |  |  |  | ☐ Horse………………………………………...9 | |  |
|  |  |  |  |  | ☐ Wild animal……………….………….…10 | |  |
|  |  |  |  |  | ☐ Other animal (specify)………….….11 | |  |
|  |  |  |  |  | …………………………….………..………………… | |  |
|  |  |  |  |  |  |  |  |
|  |  |  |  |  |  | Page **7** of **14** | |

**
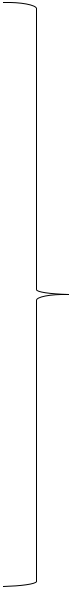
**

| **No.** | **Question** | **Interviewer’s** | |  | **Patient’s response** | | | | | **Go To** |
| --- | --- | --- | --- | --- | --- | --- | --- | --- | --- | --- |
|  |  | **Observation** | |  |  |  |  |  |  |  |
| D06 | If the exposure was due | Please tick ( | ✓ | ) one: | Please tick ( | ✓ | | | |  |
|  | to pet dog or cat, did |  |  |  |  | ) one: | | | |  |
|  |  | ☐ Yes |  |  | ☐ Vaccinated…………….……………………1 | | | | |  |
|  | the clinician ask about |  |  |  |  |  |  |  |  |  |
|  | its vaccination status? | ☐ No |  |  | ☐ Not vaccinated…………………….……..2 | | | | |  |
|  |  |  |  |  |  |  |  |  |  |  |
|  |  |  |  |  | ☐ Don’t know…………………………………3 | | | | |  |
|  |  |  |  |  |  |  |  |  |  |  |
| D07 | If vaccinated in D6, did | Please tick ( | ✓ | ) one. | Please tick ( | ✓ | | | |  |
|  | the clinician ask when |  |  |  |  | ) one. | | | |  |
|  |  | ☐ Yes |  |  | ☐ <1 year……………….………………..……..1 | | | | |  |
|  | the last date of |  |  |  |  |  |  |  |  |  |
|  | vaccination was? | ☐ No |  |  | ☐ 1-2 years……………………………………..2 | | | | |  |
|  |  |  |  |  |  |  |  |  |  |  |
|  |  |  |  |  | ☐ >2 years………………………………....…..3 | | | | |  |
|  |  |  |  |  | ☐ Don’t know ………..………………………4 | | | | |  |
|  |  |  |  |  |  |  |  |  |  |  |
| D08 | Did the clinician ask | Please tick ( | ✓ | ) one: | Please tick ( | ✓ | | | |  |
|  | about the site of direct |  |  |  |  | ) one: | | | |  |
|  |  | ☐ Yes |  |  | ☐ Head & Neck…………………..…………..1 | | | | |  |
|  | exposure on the |  |  |  |  |  |  |  |  |  |
|  | patient’s body? | ☐ No |  |  | ☐ Upper limb……………………….……..….2 | | | | |  |
|  |  |  |  |  |  |  |  |  |  |  |
|  | ***(Skip in cases of indirect*** |  |  |  | ☐ Lower limb……….…………………………3 | | | | |  |
|  | ***exposures)*** |  |  |  |  |  |  |  |  |  |
|  |  |  |  |  |  |  |  |  |  |  |
|  |  |  |  |  | ☐ Trunk……………………..………...………..4 | | | | |  |
|  |  |  |  |  | ☐ Genitalia……………………..……………..5 | | | | |  |
|  |  |  |  |  |  |  |  |  |  |  |
| D09 | Did the clinician ask | Please tick ( | ✓ | ) one: | Please tick ( | ✓ | | | |  |
|  | whether the |  |  |  |  | ) one: | | | |  |
|  |  | ☐ Yes |  |  |  |  |  |  |  |  |
|  | bite/scratch was a |  |  |  | ☐ Provoked……..…………….……...........1 | | | | |  |
|  | provoked or | ☐ No |  |  |  |  |  |  |  |  |
|  | unprovoked? |  |  |  | ☐ Unprovoked …………………….…..…..2 | | | | |  |
|  |  |  |  |  |  |  |  |  |  |  |
|  | (***Provoked act refers to*** |  |  |  |  |  |  |  |  |  |
|  |  |  |  |  |  |  |  |  |  |  |
|  | ***playing, feeding,*** |  |  |  |  |  |  |  |  |  |
|  | ***touching its offspring,*** |  |  |  |  |  |  |  |  |  |
|  | ***running in close*** |  |  |  |  |  |  |  |  |  |
|  | ***proximity, handling*** |  |  |  |  |  |  |  |  |  |
|  | ***injured animals etc***) |  |  |  |  |  |  |  |  |  |
| D10 | If the exposure was an | Please tick ( ^✓^) one: | | | Provide number or check “Unknown” | | | | |  |
|  | animal bite, did the | ☐ Yes |  |  | ☐ Number...………………………..………….1 | | | | |  |
|  | clinician ask how many |  |  |  |  |  |  |  |  |  |
|  | other people had been | ☐ No |  |  |  |  |  |  |  |  |
|  | bitten by the animal in |  |  |  |  |  |  |  |  |  |
|  |  |  |  |  | ☐ Unknown…………………………….……...2 | | | | |  |
|  | the past 1 or 2 days? |  |  |  |  |  |  |  |  |  |
|  |  |  |  |  |  |  |  |  |  |  |
|  |  |  |  |  |  |  |  |  | Page **8** of **14** | |

**
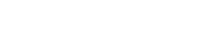
**

| **No.** | **Question** | **Interviewer’s** | |  |  | **Patient’s response** | | | | **Go To** |
| --- | --- | --- | --- | --- | --- | --- | --- | --- | --- | --- |
|  |  | **Observation** | |  |  |  |  |  |  |  |
| D11 | Did the clinician ask | Please tick ( | ✓ | ) one: |  | Please tick ( | ✓ | | |  |
|  | whether the behaviour |  |  |  |  |  | ) one: | | |  |
|  |  | ☐ Yes |  |  |  | ☐ Normal…………………………….…………1 | | | |  |
|  | of the animal involved |  |  |  |  |  |  |  |  |  |
|  | was normal or | ☐ No |  |  |  | ☐ Abnormal behaviour (with signs | | | |  |
|  | abnormal? |  |  |  |  |  |  |  |  |  |
|  |  |  |  |  |  |  |  |  |  |  |
|  |  |  |  |  |  | like aggression, excessive | | | |  |
|  |  |  |  |  |  | salivation, indiscriminate biting)….2 | | | |  |
|  |  |  |  |  |  |  |  |  |  |  |
| D12 | Did the clinician ask | Please tick ( | ✓ | ) one: |  | Please tick ( | ✓ | | |  |
|  | whether the animal |  |  |  |  |  | ) one: | | |  |
|  |  | ☐ Yes |  |  |  | ☐ Confirmed……………………...………….1 | | | |  |
|  | involved was suspected |  |  |  |  |  |  |  |  |  |
|  | or confirmed for rabies? | ☐ No |  |  |  | ☐ Suspected……………………..……..……2 | | | |  |
|  |  |  |  |  |  | ☐ Don’t know…………….….………….….3 | | | | **D14** |
|  |  |  |  |  |  |  |  |  |  |  |
| D13 | Did the clinician ask the | Please tick ( | ✓ | ) one: |  | Please tick ( | ✓ | | |  |
|  | patient about who |  |  |  |  |  | ) one: | | |  |
|  |  | ☐ Yes |  |  |  | ☐ Veterinary officials…….……………....1 | | | |  |
|  | confirmed or suspected |  |  |  |  |  |  |  |  |  |
|  | rabies in the animal? | ☐ No |  |  |  | ☐ Medical staffs………….……………..….2 | | | |  |
|  |  |  |  |  |  |  |  |  |  |  |
|  |  |  |  |  |  | ☐ Public suspicion……………………...….3 | | | |  |
|  |  |  |  |  |  | ☐ Self-suspicion……………………………..4 | | | |  |
|  |  |  |  |  |  | ☐ Other (specify)…………………….……..5 | | | |  |
|  |  |  |  |  |  | …………………………………………………………. | | | |  |
| D14 | Did the clinician ask | Please tick ( | ✓ | ) one: |  | Please tick ( | ✓ | | |  |
|  | whether the animal |  |  |  |  |  | ) one: | | |  |
|  |  | ☐ Yes |  |  |  | ☐ Yes………………………………………….….1 | | | |  |
|  | involved is still available |  |  |  |  |  |  |  |  |  |
|  | for observation? | ☐ No |  |  |  | ☐ No………………………………………….…..2 | | | |  |
|  |  |  |  |  |  |  |  |  |  |  |
|  |  |  |  |  |  |  | | | |  |
| D15 | If yes to D14, how many | Please tick ( | ✓ | ) one: |  | Please specify the number of days | | | |  |
|  | days did the clinician |  |  |  |  | advised for observation: | | | |  |
|  |  | ☐ Yes |  |  |  |  |  |  |  |  |
|  | ask the patient to |  |  |  |  |  |  |  |  |  |
|  | observe the animal? | ☐ No |  |  |  |  |  |  |  |  |
|  |  |  |  |  |  |  |  |  |  |  |
|  |  |  |  |  |  |  |  |  |  |  |


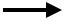


**Page 9 of** 14

| **No.** | **Question** | **Interviewer’s** | |  |  | **Patient’s response** | | | | **Go To** |
| --- | --- | --- | --- | --- | --- | --- | --- | --- | --- | --- |
|  |  | **Observation** | |  |  |  |  |  |  |  |
| D16 | Did the clinician ask | Please tick ( | ✓ | ) one: |  | Please tick ( | ✓ | | |  |
|  | whether the patient has |  |  |  |  |  | ) one: | | |  |
|  |  | ☐ Yes |  |  |  | ☐ Pre-exposure prophylaxis….……….1 | | | |  |
|  | received rabies pre/post |  |  |  |  |  |  |  |  |  |
|  | exposure prophylaxis | ☐ No |  |  |  | ☐ Post-exposure prophylaxis (IM).…2 | | | |  |
|  | prior to this exposure? |  |  |  |  |  |  |  |  | **D18** |
|  |  |  |  |  |  |  |  |  |  |  |
|  |  |  |  |  |  | ☐ Post-exposure prophylaxis (ID)…..3 | | | |  |
|  |  |  |  |  |  |  |  |  |  |  |
|  |  |  |  |  |  | ☐ None…………………………………………..4 | | | | **D21** |
|  |  |  |  |  |  |  |  |  |  |  |
| D17 | If yes to pre-exposure | Please tick ( | ✓ | ) one: |  | Please tick ( | ✓ | | |  |
|  | prophylaxis, did the |  |  |  |  |  | ) one or more: | | |  |
|  |  | ☐ Yes |  |  |  | ☐ Day 0……………………………….….……..1 | | | |  |
|  | clinician ask whether |  |  |  |  |  |  |  |  |  |
|  | the patient has received | ☐ No |  |  |  | ☐ Day 7……………………….…………………2 | | | |  |
|  | complete course or not? |  |  |  |  |  |  |  |  |  |
|  |  |  |  |  |  | ☐ Day 28………………….…………………….3 | | | |  |
|  |  |  |  |  |  |  |  |  |  |  |
| D18 | If yes to post-exposure | Please tick ( | ✓ | ) one: |  | Please tick ( | ✓ | | |  |
|  | prophylaxis (IM), did the |  |  |  |  |  | ) one or more: | | |  |
|  |  | ☐ Yes |  |  |  | ☐ Day 0………………………….……….……..1 | | | |  |
|  | clinician ask whether |  |  |  |  |  |  |  |  |  |
|  | the patient has received | ☐ No |  |  |  | ☐ Day 3……………………….…………...…...2 | | | |  |
|  | complete course or not? |  |  |  |  |  |  |  |  |  |
|  |  |  |  |  |  | ☐ Day 7………………….……………….…....3 | | | |  |
|  |  |  |  |  |  | ☐ Day 14………………….……………..……..4 | | | |  |
|  |  |  |  |  |  | ☐ Day 28………………………………….….…5 | | | |  |
|  |  |  |  |  |  |  |  |  |  |  |
| D19 | If yes to post-exposure | Please tick ( | ✓ | ) one: |  | Please tick ( | ✓ | | |  |
|  | prophylaxis (ID), did the |  |  |  |  |  | ) one or more: | | |  |
|  |  | ☐ Yes |  |  |  | ☐ Day 0………………………………….….…..1 | | | |  |
|  | clinician ask whether |  |  |  |  |  |  |  |  |  |
|  | the patient has received | ☐ No |  |  |  | ☐ Day 3…………………….…..…….………...2 | | | |  |
|  | complete course or not? |  |  |  |  |  |  |  |  |  |
|  |  |  |  |  |  | ☐ Day 7………………………………….….....3 | | | |  |
|  |  |  |  |  |  | ☐ Day 28……………………………………..…4 | | | |  |
|  |  |  |  |  |  |  | | | |  |
| D20 | If yes to pre/post | Please tick ( | ✓ | ) one: |  | Please specify how long ago in | | | |  |
|  | prophylaxis, did the |  |  |  |  | months: |  |  |  |  |
|  |  | ☐ Yes |  |  |  |  |  |  |  |  |
|  | clinician ask how long |  |  |  |  |  |  |  |  |  |
|  | ago was the last | ☐ No |  |  |  |  |  |  |  |  |
|  | vaccination? |  |  |  |  |  |  |  |  |  |
|  |  |  |  |  |  |  |  |  |  |  |


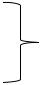

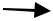


**Page 10 of** 14

| **No.** |  | **Question** | **Interviewer’s** | | |  | **Patient’s response** | | | | | **Go To** | |
| --- | --- | --- | --- | --- | --- | --- | --- | --- | --- | --- | --- | --- | --- |
|  |  |  | **Observation** | | |  |  |  |  |  |  |  |  |
| D21 |  | Did the clinician ask | Please tick ( | ✓ | | ) one: | Please tick ( | | ✓ | | |  |  |
|  |  | whether patient has |  |  |  |  |  |  |  | ) one: | |  |  |
|  |  |  | ☐ Yes |  |  |  | ☐ Running water………………..………….1 | | | | |  |  |
|  |  | washed the wound |  |  |  |  |  |  |  |  |  |  |  |
|  |  | immediately after the | ☐ No |  |  |  | ☐ Running water with soap/ | | | | |  |  |
|  |  | direct exposure? |  |  |  |  |  |  |  |  |  |  |  |
|  |  |  |  |  |  |  |  |  |  |  |  |  |  |
|  |  |  |  |  |  |  | detergent…………………………………..2 | | | | |  |  |
|  |  |  |  |  |  |  | ☐ Didn’t wash the wound ….………….3 | | | | |  |  |
|  |  |  |  |  |  |  |  |  |  |  |  |  |  |
| D22 |  | If yes to D21, did the | Please tick ( | ✓ | | ) one: | Please tick ( | | ✓ | ) one: | |  |  |
|  |  | clinician ask for how |  |  |  |  |  |  |  |  |  |  |  |
|  |  |  | ☐ Yes |  |  |  | ☐ 1 minute………………………..…………..1 | | | | |  |  |
|  |  | long the patient washed |  |  |  |  |  |  |  |  |  |  |  |
|  |  | the wound? | ☐ No |  |  |  | ☐ 2-4 minutes…………………..……………2 | | | | |  |  |
|  |  |  |  |  |  |  |  |  |  |  |  |  |  |
|  |  |  |  |  |  |  | ☐ 5-9 minutes………………..………….….3 | | | | |  |  |
|  |  |  |  |  |  |  | ☐ ≥10 minutes………………………………4 | | | | |  |  |
|  |  |  |  |  |  |  |  |  |  |  |  |  |  |
| D23 |  | Did the clinician ask | Please tick ( | ✓ | | ) one: | Please tick ( | | ✓ | ) one: | |  |  |
|  |  | whether the patient |  |  |  |  |  |  |  |  |  |  |  |
|  |  |  | ☐ Yes |  |  |  | ☐ Yes……………………………………..….…..1 | | | | |  |  |
|  |  | applied any antiseptic |  |  |  |  |  |  |  |  |  |  |  |
|  |  | to wound or not? | ☐ No |  |  |  | ☐ No…………………..…………………..….….2 | | | | |  |  |
|  |  |  |  |  |  |  |  |  |  |  |  |  |  |
|  |  |  |  |  |  |  |  |  |  |  |  |  |  |
|  |  | **E. Clinician’s Decision** | |  |  |  |  |  |  |  |  |  |  |
|  |  |  |  |  |  |  | | |  |  |  | |  |
| **No.** |  | **Question** |  |  |  | **Clinician’s decision** | | |  |  | **Go To** | |  |
|  | |  | | |  |  |  |  |  |  |  |  |  |
| E01 | | Did the clinician categorise the type of | | |  | Please tick ( | | ✓ |  |  |  |  |  |
|  |  | exposure? |  |  |  |  |  | ) one: | |  |  |  |  |
|  |  |  |  |  |  | ☐ Yes……………………………….........................1 | | | | |  |  |  |
|  |  |  |  |  |  |  |  |  |  |  |  |  |  |
|  |  |  |  |  |  | ☐ No…………………………….……………….……..…2 | | | | |  | **E03** |  |
|  | |  | |  |  |  |  |  |  |  |  |  |  |
| E02 | | If yes, what was the category of | |  |  | Please tick ( | | ✓ |  |  |  |  |  |
|  |  | exposure assigned by the clinician? | |  |  |  |  | ) one: | |  |  |  |  |
|  |  |  |  |  |  | ☐ No category…………………………….....….…..1 | | | | |  |  |  |
|  |  |  |  |  |  |  |  |  |  |  |  |  |  |
|  |  |  |  |  |  | ☐ Category I…………………………..……..…….….2 | | | | |  |  |  |
|  |  |  |  |  |  | ☐ Category II…………………….………..…..........3 | | | | |  |  |  |
|  |  |  |  |  |  | ☐ Category III………………….………………….…..4 | | | | |  |  |  |
|  |  |  |  |  |  |  |  |  |  |  |  | |  |
|  |  |  |  |  |  |  |  |  |  | Page **11** of **14** | | | |

**
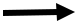
**

| **No.** | **Question** | **Clinician’s decision** | | **Go To** |
| --- | --- | --- | --- | --- |
|  |  |  |  |  |
| E03 | What treatment did the clinician | Please tick ( | ✓ |  |
|  | prescribe? |  | ) one or more: |  |
|  |  | ☐ ARV (ID)………………………..……….……………1 | |  |
|  |  |  |  |  |
|  |  | ☐ ARV (IM)…………………………...……..……...…2 | |  |
|  |  | ☐ RIG……………….........................................3 | |  |
|  |  | ☐ Tetanus toxoid………………..….…………..….4 | |  |
|  |  | ☐ Antibiotics……………………….………..…..…...5 | |  |
|  |  | ☐ Wound suturing……………..……………………6 | |  |
|  |  | ☐ Wound dressing……………..….…………….…7 | |  |
|  |  | ☐ Reassurance………………………………….….…8 | |  |
|  |  | ☐ Other treatment (specify)……………………9 | |  |
|  |  | ………………………………………………...…………....... | |  |
| E04 | If wound washing is prescribed, what is | Please tick ( ^✓^) one: | |  |
|  | the wound washing method prescribed | ☐ Running water………………….…..………..…..1 | |  |
|  | by the clinician? |  |  |  |
|  |  | ☐ Running water with soap/detergent.…..2 | |  |
|  |  | ☐ Didn’t prescribe…………………….……...……3 | | **E06** |
|  |  |  |  |  |
|  |  |  |  |  |
| E05 | If wound washing is prescribed, did | Please tick ( | ✓ |  |
|  | clinician specify the duration of wound |  | ) one: |  |
|  |  | ☐ 1 minute…………………………………………..…1 | |  |
|  | washing? |  |  |  |
|  |  | ☐ 2-5 minutes…….…….…………………………….2 | |  |
|  |  | ☐ 6-9 minutes………….…….……………………….3 | |  |
|  |  | ☐ ≥10 minutes……………….……..……………….4 | |  |
|  |  |  |  |  |


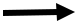


**Page 12 of** 14

| 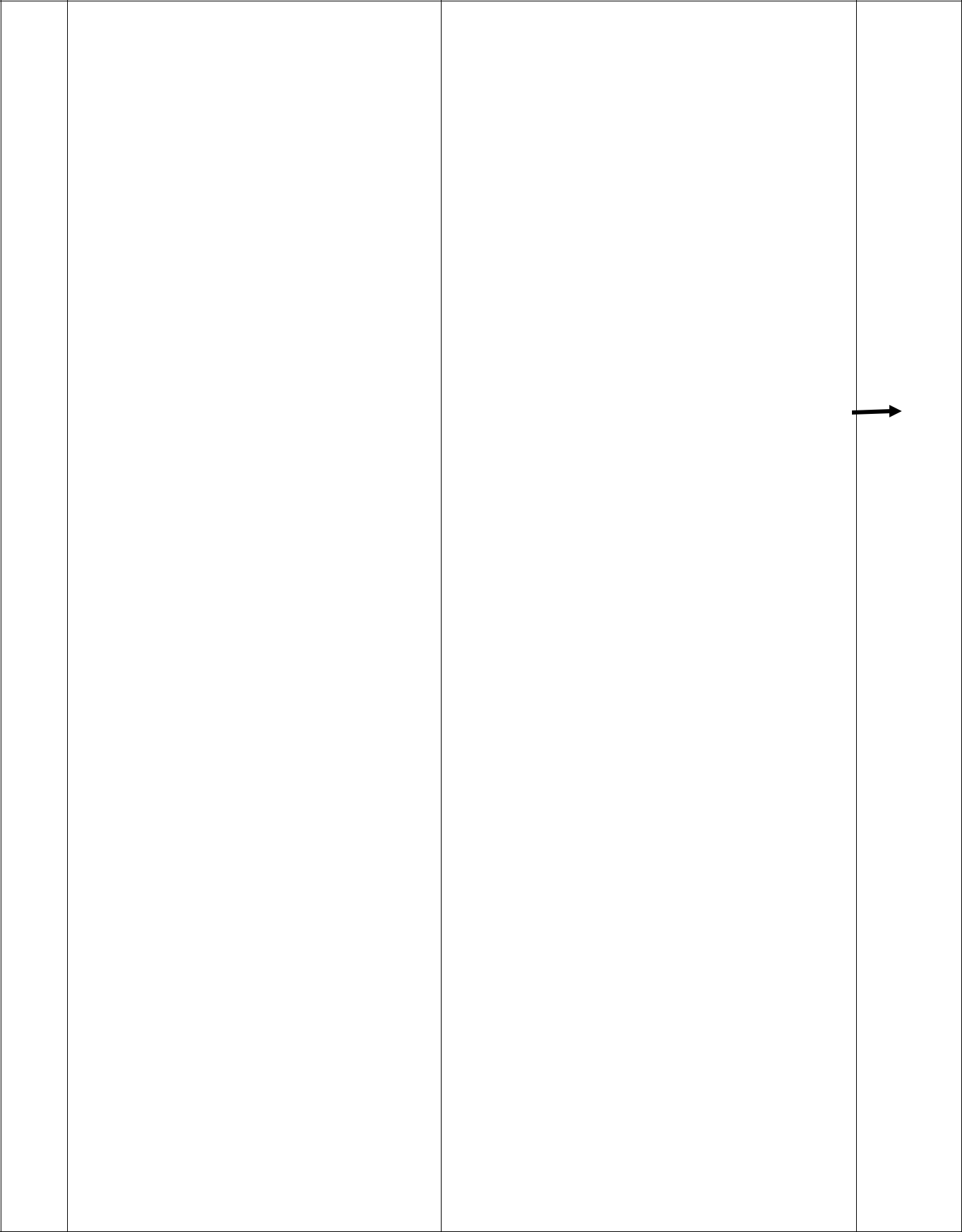**No. Question** | **Clinician’s decision** | | **Go To** |
| --- | --- | --- | --- |
|  |  | |  |
| E06 What antiseptic did the clinician advise | Please tick ( ^✓^) one: | |  |
| for application to the wound? | ☐ Povidone iodine…………..………….….………1 | |  |
|  |  |  |  |
|  | ☐ Alcohol……………..…….…..….……….….……..2 | |  |
|  | ☐ Others………………………..…..……….….….....3 | |  |
|  | ☐ None……………………………..…….…..….……..4 | |  |
|  |  | |  |
| E07 Did the clinician give counselling to the | ✓ | |  |
| patient? | Please tick ( | ) one: |  |
|  | ☐ Yes……………….………….………….…………...…1 | |  |
|  |  |  |  |
|  | ☐ No…………………….……….…..…….…………....2 | | **E09** |
|  |  |  |  |
| E08 If yes, what was the advice given by | Please tick ( | ✓ |  |
| the clinician? |  | ) one or more: |  |
|  | ☐ Observe dogs/cats for 10 days for | |  |
|  |  |  |  |
|  | clinical signs of rabies …………….…………...1 | |  |
|  | ☐ If an animal exhibits clinical signs of | |  |
|  | rabies or dies during the observation | |  |
|  | period, patient should report to hospital | |  |
|  | immediately……………………….….……..……2 | |  |
|  | ☐ Report to veterinary official to seek | |  |

advice on potential signs of rabies in

animal……………………..…………….………....3

- If an animal is normal during

observation period, report to the

hospital……………….……………….…………...4

- Advise to complete full PEP………..………5
- Advise about wound management at

home……..……………………………………………6

- Avoid alcohol drinking…….………………....7
- Others (specify)..…………….………….….....8

…………………………………………………………………

**Page 13 of** 14

| **No.** | **Question** | **Clinician’s decision** | | **Go To** |
| --- | --- | --- | --- | --- |
|  |  |  |  |  |
| E09 | If ARV was prescribed by the clinician | Please tick ( | ✓ |  |
|  | has the patient received the complete |  | ) one or more: |  |
|  |  | ☐ Day 0…………………………………………….…....1 | |  |
|  | PEP? |  |  |  |
|  | *(To be answered through follow up by* | ☐ Day 3………………………………..……..………....2 | |  |
|  | *telephone call at the end of one month* | ☐ Day 7………………………….….…….…….……...3 | |  |
|  | *or through ARV treatment register)* |  |  |  |
|  |  |  |  |  |
|  |  | ☐ Day 14……………………………..……..…………..4 | |  |
|  |  | ☐ Day 28………………………………….……..………5 | |  |
|  |  | ☐ Wasn’t prescribed……………………………….6 | |  |
|  |  |  |  |  |
| E10 | What is the risk of rabies exposure | Please tick ( | ✓ |  |
|  | assessed in the patient? |  | ) one or more: |  |
|  |  | ☐ Suspected……………..……………..……………..1 | |  |
|  |  |  |  |  |
|  |  | ☐ Confirmed clinically…………………………….2 | |  |
|  |  | ☐ None………………………..…….…….……...…....3 | |  |
|  |  |  |  |  |
|  | If rabies was suspected or clinically | Please tick ( | ✓ |  |
| E11 | confirmed in the animal, what are the |  | ) one or more: |  |
|  |  | ☐ The sample from animal was tested by | |  |
|  | reasons or basis for it? |  |  |  |
|  |  | laboratory and confirmed…………...……..1 | |  |
|  |  | ☐ The animal showed typical signs of | |  |
|  |  | rabies like aggression and attacking | |  |
|  |  | people, paralysis, excessive salivation or | |  |
|  |  | other abnormal behaviour………………....2 | |  |
|  |  | ☐ The animal had died within 10 days…....3 | |  |
|  |  | ☐ Other reason (Specify)………………....…….4 | |  |
|  |  | …………………………………………………..……….. | |  |

**Any other general note by the trained health staffs in the clinician chamber:**

***This is the end of our survey!***

Page **14** of **14**
